# The Role of MKP-1 in the Anti-Proliferative Effects of Glucocorticoids in Primary Rat Pre-Osteoblasts

**DOI:** 10.1371/journal.pone.0135358

**Published:** 2015-08-11

**Authors:** Micheline Sanderson, Hanél Sadie-Van Gijsen, Stephen Hough, William F. Ferris

**Affiliations:** Division of Endocrinology, Department of Medicine, Faculty of Medicine and Health Sciences, Stellenbosch University Tygerberg Campus, Parow, South Africa; Second University of Naples, ITALY

## Abstract

Glucocorticoid (GC)-induced osteoporosis has been attributed to a GC-induced suppression of pre-osteoblast proliferation. Our previous work identified a critical role for mitogen-activated protein kinase (MAPK) phosphatase-1 (MKP-1) in mediating the anti-proliferative effects of GCs in immortalized pre-osteoblasts, but we subsequently found that MKP-1 null mice were not protected against the pathological effects of GCs on bone. In order to reconcile this discrepancy, we have assessed the effects of GCs on proliferation, activation of the MAPK ERK1/2 and MKP-1 expression in primary adipose-derived stromal cells (ADSCs) and ADSC-derived pre-osteoblasts (ADSC-OBs). ADSCs were isolated by means of collagenase digestion from adipose tissue biopsies harvested from adult male Wistar rats. ADSC-OBs were prepared by treating ADSCs with osteoblast differentiation media for 7 days. The effects of increasing concentrations of the GC dexamethasone on basal and mitogen-stimulated cell proliferation were quantified by tritiated thymidine incorporation. ERK1/2 activity was measured by Western blotting, while MKP-1 expression was quantified on both RNA and protein levels, using semi-quantitative real-time PCR and Western blotting, respectively. GCs were strongly anti-proliferative in both naïve ADSCs and ADSC-OBs, but had very little effect on mitogen-induced ERK1/2 activation and did not upregulate MKP-1 protein expression. These findings suggest that the anti-proliferative effects of GCs in primary ADSCs and ADSC-OBs *in vitro* do not require the inhibition of ERK1/2 activation by MKP-1, which is consistent with our *in vivo* findings in MKP-1 null mice.

## Introduction

Glucocorticoids (GC) are frequently used to manage diseases that result from an inappropriate inflammatory response, such as asthma, rheumatoid arthritis and inflammatory bowel syndrome. However, a serious side-effect of chronic, high-dose GC administration is decreased bone mineral density which may progress to osteoporosis [1,2] and an increased risk of bone fracture [3]. Whereas the initial rapid bone loss after GC treatment is likely to be caused by increased osteoclast activity, the progressive pathological effects of GC treatment have been attributed to a decrease in osteoblast number and function [4]. It is well established that physiological concentrations of GCs promote osteoblast differentiation and function [5]. However, the decline in the availability of functional osteoblasts in the presence of elevated (pharmacological) levels of GCs is a consequence of GC-induced inhibition of osteoblast differentiation [5] and pre-osteoblast proliferation [5,6], an increase in osteoblast apoptosis [7] and the promotion of aberrant differentiation of osteoblast progenitor cells into an adipocytic phenotype [8]. This has been demonstrated *in vitro* in mesenchymal stromal cells (MSCs), where low concentrations of GCs in the osteogenic differentiation cocktail (usually 10 nM dexamethasone) are sufficient for the progression towards a fully mature osteoblastic phenotype [9–11], while higher GC doses negatively affect proliferation [12,13] and induce an increase in triglyceride accumulation [14]. Although the molecular mechanism underlying the negative effects of GCs on pre-osteoblast mitogenesis have been investigated in immortalized early pre-osteoblast cell lines, there is a paucity of information regarding the molecular events that occur during the modulation of proliferation by GCs in primary MSCs and pre-osteoblasts.

The activation of the extracellular signal-regulated (ERK) 1/2 signalling pathway is essential for mitogenesis in a multitude of cell types [15–17], including the immortalized mouse pre-osteoblast cell line, MBA-15.4 [18]. Earlier results from our group demonstrated that high concentrations of GCs abrogate both mitogen-stimulated proliferation and the associated sustained ERK1/2 activation in this cell line. This was attributed to an increase in the activity and expression of phosphatases which dephosphorylate ERK1/2, thus switching off this mitogenic pathway. In particular, the dual-specificity phosphatase mitogen-activated protein kinase phosphatase-1 (MKP-1) has been found to be a highly dominant phosphatase that is up-regulated by GCs and inhibits ERK1/2 activity in the MBA-15.4 cell line [18,19]. As a continuation of these studies, we have recently examined the consequence of global MKP-1 knockout on the effects of pathological doses of GCs on mouse bone and found that deletion of MKP-1 expression has no effect on the ability of GCs to reduce osteoblast surfaces, bone formation rate and bone strength [20]. Consequently, as MKP-1 is unlikely to be the dominant conduit for the detrimental effects of GCs on osteoblast number and function in mouse bone, we wished to examine this disparity between the *in vivo* data obtained in mice and the *in vitro* data from MBA-15.4 cells. In addition, we sought to achieve a better understanding of the mechanism underlying the anti-proliferative effects of GCs on a cellular level. To this end, the effects of GCs on proliferation, ERK activation and MKP-1 expression were assessed in primary naïve adipose-derived mesenchymal stromal cells (ADSCs) and also in ADSCs that were partially differentiated into pre-osteoblasts (ADSC-OBs). ADSCs are more readily isolated than bone marrow-derived MSCs, can be purified to homogeneity and have *in vitro* osteoblast differentiation potential [21,22]. We have previously demonstrated that ADSCs differentiate into bone-forming osteoblasts and deposit calcified nodules after being cultured for 28 days in osteoblast differentiation media (OM) [11]. It was therefore arbitrarily taken that ADSCs cultured for 7 days in OM could be classed as pre-osteoblasts, as these cells exhibit markers of early osteoblast differentiation, including increased expression of Msx2 and alkaline phosphatase, but are not mature enough to deposit calcified extracellular matrix [11].

## Materials and Methods

### Materials

Fetal bovine serum was purchased from Invitrogen, Paisley, UK and Penicillin / Streptomycin and trypsin solutions from Lonza, Walkersville, MD, USA. Sodium pentobarbitone was obtained from Bayer, Isando, South Africa and collagenase I (#CLS1) from Worthington, Lakewood, NJ, USA. Tritiated thymidine ([^3^H]dT, #TRK 120), horseradish peroxidase (HRP)-linked anti-rabbit IgG secondary antibody (from donkey, #NA934) and Hyperfilm ECL high performance ECL film were all purchased from General Electric Healthcare (Buckinghamshire, UK). Primary anti-p44/42 MAPK (#9102) and anti-phospho-p44/42 MAPK (#9101) antibodies were purchased from Cell Signaling Technology (Beverly, MA, USA), whereas the primary MKP-1 (#sc-1102) antibody was from Santa Cruz Biotechnology (Santa Cruz, CA, USA). LumiGLO reserve chemiluminescent substrate kit was purchased from KPL laboratories (Gaithersburg, Maryland, USA). Primers were produced by Integrated DNA Technologies Incorporated (IDT) and Metabion (Roche Diagnostics Corporation, IN, USA). Biotrace PVDF membranes were purchased from Pall Life Sciences, Pall Corporation, Florida, USA. All other reagents, including Dulbecco’s Modified Eagle Medium (DMEM) were purchased from Sigma, Louisiana, USA.

### Experimental animals

All experiments involving animals were performed in accordance with the South African Medical Research Council Guidelines on Ethics for Medical Research and were approved by the Stellenbosch University ethics committee. This complies with the South African Animal Protection Act (Act No. 71 of 1962). Animals were fed *ad libitum* on standard laboratory chow.

### Isolation of rat ADSCs

Male Wistar rats (28 weeks old) were terminated by lethal injection with sodium pentobarbitone (12 mg/kg) and inguinal subcutaneous adipose tissue (approximately 3–5 cm^3^) excised. ADSCs were isolated and purified by means of collagenase digestion [22], with modifications as previously published [23].

### Cell culture and osteoblast differentiation

All cells were grown and maintained in 95% humidified air with 5% CO_2_ at 37°C. Cells were cultured in high-glucose DMEM containing 10% fetal bovine serum (FBS) and 1% penicillin/streptomycin. All experiments were performed at passage 2. For culture expansion, cells at 80% confluence were treated with 0.5% trypsin solution and subcultured at a dilution of 1:5. For the generation of ADSC-OBs, ADSCs were grown until confluence (day 0) and subsequently treated for 7 days with osteoblast differentiation media (OM), which consisted of standard culture media (DMEM plus 10% FBS) supplemented with 10 nM dexamethasone, 10 mM glycerol-2-phosphate and 50 μM ascorbic acid [9–11]. OM was prepared immediately prior to use, and was replaced every 2–3 days.

### Proliferation assay using [^3^H]dT incorporation

For experiments examining proliferation in naïve ADSCs, subconfluent ADSC cultures (50–70% confluent) were first made synchronous by serum starvation (DMEM containing 1% FBS) for 24 hours. This severely suppresses, but does not totally inhibit growth in these cultures, whilst maintaining viability. FBS concentrations of less than 1% resulted in substantially diminished cell viability and could not be used to further reduce background proliferation (data not shown). For experiments in ADSC-derived pre-osteoblasts (ADSC-OBs), confluent naïve ADSC cultures were treated with OM for 7 days before mitogenic stimulation was performed. The effect of mitogens and mitogenic inhibitors on proliferation was measured using [^3^H]dT incorporation. The mitogen and/or inhibitor was added to cells in 24 well plates, at a concentration as indicated in the respective figure legends, and subsequently incubated for 24 hours. Cells were labelled with 2μCi /ml of [^3^H]dT (specific activity = 25 Ci/mmol) for 4 hours prior to the end of the incubation time. The reaction was inhibited by placing the plate on ice for 2 minutes, after which media was removed from the wells and the cells washed twice with ice-cold PBS. The plate was then placed at -80°C for 1 hour to aid subsequent cell lysis, after which the cells were thawed at room temperature (RT) before the addition of 500 μl of lysis solution (0.1% SDS, 0.1% NaOH) and incubation at RT for 1 hour. An additional 350 μl of lysis solution was added to each sample, and acid-insoluble DNA and proteins were precipitated by adding 500 μl of ice-cold 50% (w/v) TCA (trichloroacetic acid) to the cell lysates, followed by overnight incubation at 4°C. DNA pellets were collected by centrifugation at 15 000 x g for 30 min at 4°C. Precipitates were washed with 500 μl of 10% (w/v) ice-cold TCA, after which pellets were collected and resuspended in 500 μl of 0.1 N NaOH and incubated at RT for 1 hour with intermittent vortex mixing. For the quantification of [3H]dT incorporated into DNA, 8 ml of Ready-gel scintillation fluid (Beckman Coulter) was added to 350 μl of each sample and clarified by the addition of 600 μl of glacial acetic acid. Radioactive decay was determined on a LS5000TD scintillation counter (Beckman Coulter) for 5 minutes per sample, and results are displayed as counts per minute (cpm).

### Protein extraction and Western blot analysis

For the preparation of protein extracts, naïve ADSCs were plated in 100mm cell culture petri dishes and grown to 80% confluence. Alternatively, cells were grown to confluence and treated for 7 days with OM to generate pre-osteoblasts (ADSC-OBs). Cell cultures were treated as described in the relevant figure legends, after which the culture dishes were immediately placed on ice. Cells were washed twice in ice-cold PBS and treated with lysis buffer (0.1% Triton X-100, 50 mM Tris, 120 mM NaCl, 5 mM EDTA, 1 mM EGTA, 5 mM NaF, 20 μM β-glycerophosphate, 2 mM Na3VO4, 1 mM PMSF, 10 μg/ml leupeptin, 10 μg/ml aprotinin, 10 μg/ml pepstatin, pH 7.5). Cell suspensions were sonicated for 10 seconds (at an amplitude of 20 microns peak to peak) and debris pellets collected at 15 000 x g at 4°C for 10 minutes. Supernatant fractions were transferred to pre-chilled 2 ml microfuge tubes for subsequent use as protein cell lysates. Protein concentrations were determined by means of the Bradford assay [24].

For Western blot analysis, either 15 μg (for ERK 1/2 detection) or 70 μg (for MKP-1 detection) of total lysate protein was separated and resolved by electrophoresis on a SDS-PAGE gel [25]. Proteins were transferred onto PVDF membranes at 30 V overnight with cooling. After protein transfer, membranes were briefly washed in 100% methanol and dried for 15 min to enhance protein binding. Protein loading and transfer efficiency were assessed by staining membranes with Ponceau S solution (0.2% Ponceau S in 5% glacial acetic acid). Membranes were blocked for 1 hour at RT with blocking solution (5% fat-free milk powder, 0.1% Tween 20 in TBS), and washed three times for 10 minutes with TBS-Tween (0.1% Tween 20 in TBS) before overnight incubation with primary antibodies at 4°C. Anti-ERK and anti-phospho-ERK antibodies were used at 1/1000 dilution, while the anti-MKP-1 antibody was used at 1/500 dilution. Prior to incubation with HRP-conjugated secondary antibodies, at a dilution of 1/4000, membranes were washed three times for 10 minutes with TBS-Tween. Immuno-responsive protein bands were visualised after incubation with LumiGlo solution, exposure of the membranes to Hyperfilm ECL and photographic development of the film. Films were scanned on a Microtek ScanMaker 8700 using ScanWizard Pro software (Microtek International Inc., Taiwan) and quantification of immuno-responsive bands was performed with ImageJ image analysis software.

### RNA isolation and real-time semiquantitative RT-PCR

Total RNA was isolated using the Promega SV Total RNA isolation system. Any contaminating DNA was then removed by digesting 1 μg total RNA with RNase-free DNase (Promega RQ1, as per manufacturer’s instructions). cDNA was synthesised from this DNase-treated RNA using a 20mer poly-dT primer and Promega ImProm-II reverse transcriptase. Real-time semi-quantitative PCR was performed on a Rotor-Gene 6000 (Corbett Life Science) using a Quantace Sensimix kit. The ΔCt method [26] was used to calculate gene expression levels relative to the housekeeping gene, acidic ribosomal phosphoprotein (ARBP) [27]. Primers for ARBP and MKP-1 [28] are given in Table 1.

**Table 1:** Primer sequences used in real-time PCR.

Transcript	Forward primer	Reverse primer	Product size
Rat MKP-1 [28]	CTGCTTTGATCAACGTCTCG	AAGCTGAAGTTGGGGGAGAT	301
Rat ARBP [27]	AAAGGGTCCTGGCTTTGTCT	GCAAATGCAGATGGATCG	91

### Statistical analysis

Unless otherwise stated in the figure legends, all results were graphically represented and statistically analysed using one-, two- or three-way ANOVA and Bonferroni’s post-test where appropriate. Statistically significant differences (p<0.05) between values are indicated with different lower-case letters (e.g. a vs b) above each bar.

## Results

### FBS stimulates proliferation in naïve ADSCs and in ADSC-OBs via a U0126-sensitive mechanism

**Fig 1:**
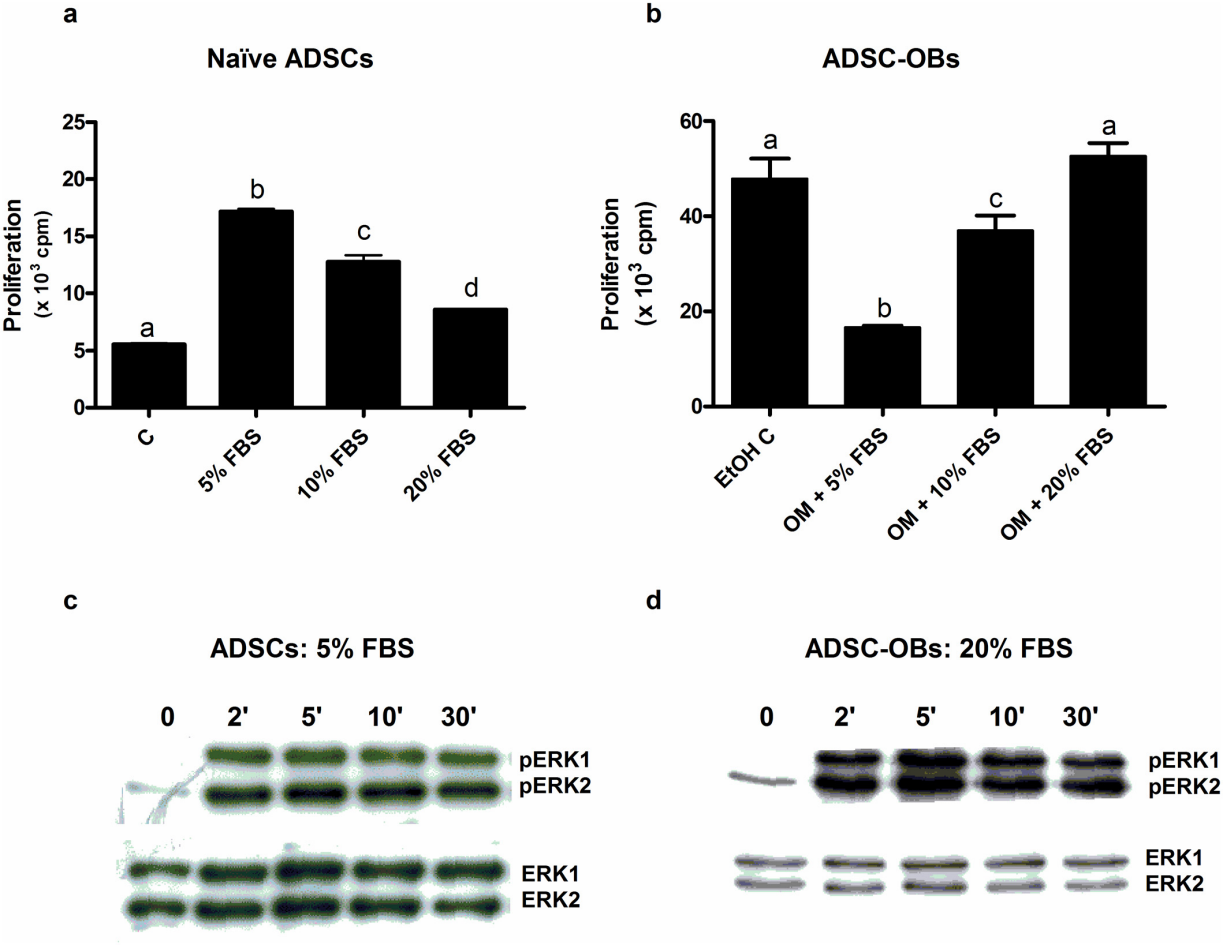
Stimulation of proliferation and ERK1/2 activation by FBS. (a): Naïve ADSCs were plated in 24-well plates and grown to 80% confluence, after which they were cultured in DMEM + 1% FBS for 24h. Cells were subsequently treated for 24h with DMEM supplemented with FBS at the concentrations given, and proliferation was quantified by means of [^3^H]dT incorporation. (b): Naïve ADSCs were plated in 24-well plates and grown to 100% confluence, upon which they were treated for 7 days with vehicle control (0.1% EtOH) or with OM to generate ADSC-OBs, which were subsequently treated for 24h with OM supplemented with FBS at the concentrations indicated. Proliferation was quantified as for (a). (c,d): Western blotting for total ERK1/2 and phospho-ERK1/2 was subsequently performed on cell lysates. (c): Naïve ADSCs were plated in 100mm dishes and grown to 80% confluence, after which they were cultured in DMEM + 1% FBS for 24h. Cells were subsequently treated with DMEM + 5% FBS for the time-points indicated, and cell lysates were prepared. (d): ADSC-OBs were generated as for (a) and subsequently treated with OM + 20% FBS for the time-points indicated, after which cell lysates were prepared. Panels a and b show the representative results of three independent experiments, performed in triplicate. Panels c and d show the representative results of three independent experiments. Different lower-case letters indicate statistically significant differences (P < 0.05).

In order to establish conditions of maximal mitogenic stimulation, naïve ADSCs and ADSC-OBs were treated for 24 hours with increasing concentrations of FBS. It was found that 5% FBS induced the highest rate of proliferation in naïve ADSCs, compared to 10% and 20% FBS, which stimulated proliferation to a lesser extent (Fig 1a). In contrast with the results obtained for naïve ADSCs, 20% FBS maximally stimulated proliferation in ADSC-OBs (within the context of the other constituents of OM), while 5% and 10% FBS were less effective (Fig 1b).

The activation of the ERK1/2 pathway is essential for proliferation in the majority of cells [15], including immortalized MBA 15.4 and MG63 pre-osteoblasts [18], and therefore the relationship between proliferation and ERK1/2 activity in primary naïve ADSCs and ADSC-OBs was investigated by examining ERK1/2 phosphorylation after mitogenic stimulation. Western blot analysis was used to quantify the ratio of active, phosphorylated ERK1/2 to total ERK1/2. ERK1/2 was found to be strongly and rapidly phosphorylated in naïve ADSCs after an increase of FBS in the cell culture media from 1% to 5% (Fig 1c). Similar observations were made in ADSC-OBs upon the addition of 20% FBS (Fig 1d). In the absence of mitogenic stimulation, phospho-ERK1/2 was virtually undetectable (Fig 1c and 1d).

**Fig 2:**
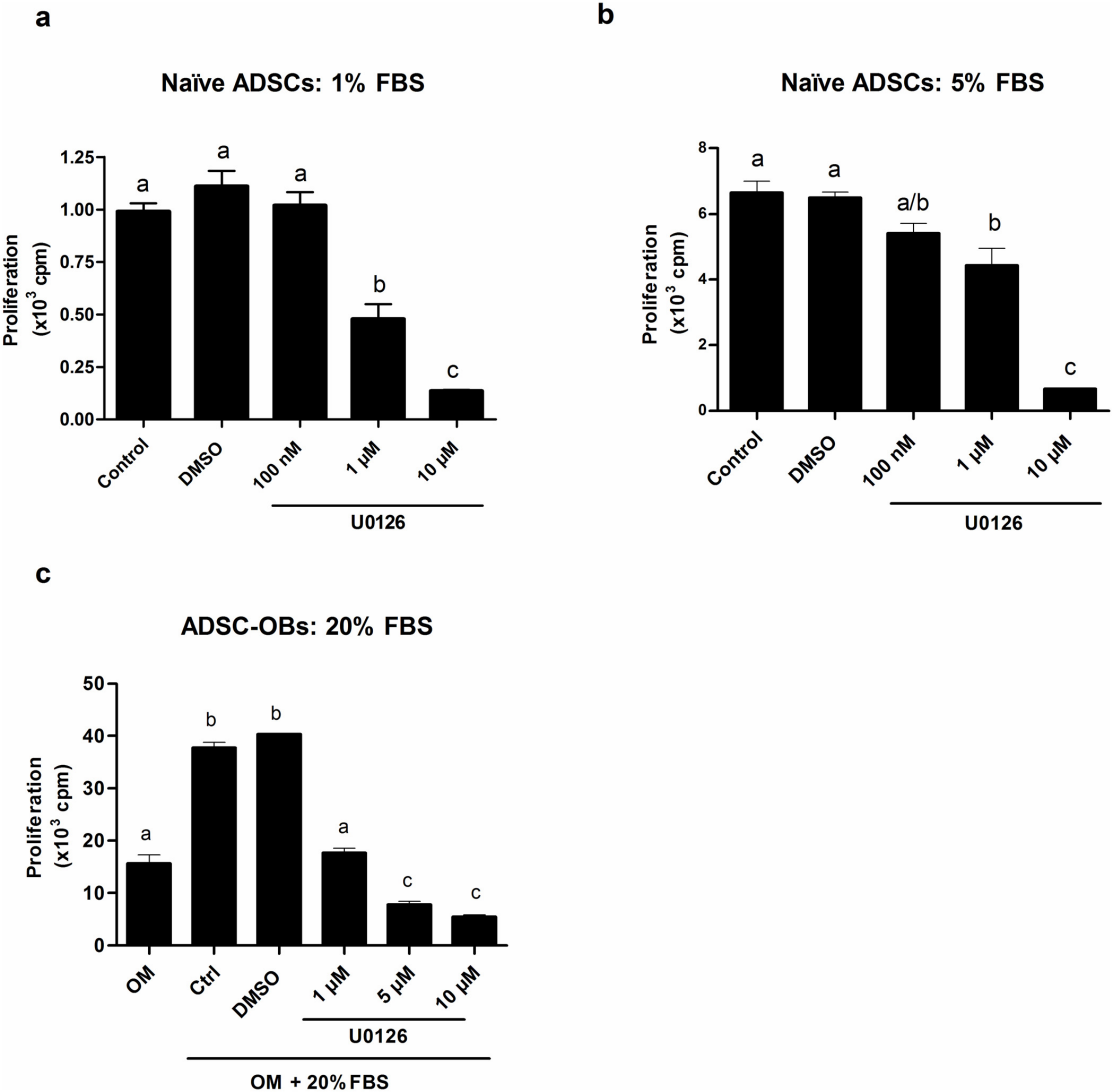
ERK1/2 activity is essential for basal and mitogen-stimulated proliferation. (a,b): Naïve ADSCs were plated in 24-well plates and grown to 80% confluence, after which they were cultured in DMEM + 1% FBS for 24h and subsequently treated with (a) DMEM + 1% FBS, maintaining basal proliferation or (b) mitogenically stimulated with DMEM + 5% FBS, in the absence (DMSO control) or presence of different concentrations of U0126, for 24h. Proliferation was quantified by means of [^3^H]dT incorporation. (c): Naïve ADSCs were plated in 24-well plates, grown to 100% confluence and treated with OM for 7 days to generate ADSC-OBs, which were subsequently mitogenically stimulated with OM + 20% FBS, in the absence (DMSO control) or presence of different concentrations of U0126, for 24h. Proliferation was quantified as for (a). Panels a-c show the representative results of three independent experiments, performed in triplicate. Different lower-case letters indicate statistically significant differences (P < 0.05).

The importance of ERK1/2 activation during ADSC and ADSC-OB proliferation was assessed by the use of the upstream MEK1/2 inhibitor U0126 [29]. In naïve ADSCs, both basal (1% FBS: Fig 2a) and mitogen-stimulated proliferation (5% FBS: Fig 2b) was inhibited in a dose-dependent manner by U0126. Similar results were observed during mitogen-stimulated proliferation (20% FBS) of ADSC-OBs (Fig 2c). These findings indicate the essential involvement of ERK1/2 in proliferation under all of these conditions. Inhibition of mitogen-stimulated ERK1/2 activation by U0126 in naïve ADSCs and ADSC-OBs was confirmed by Western blotting, which demonstrated the absence of phospho-ERK1 and phospho-ERK2 bands in the presence of U0126 (results not shown).

[3H]dT incorporation consistently (n = 5) showed that inhibition of ERK1/2 by U0126 during basal (unstimulated) ADSC growth (Fig 2a) resulted in assay counts equivalent to that of the background radioactivity, inferring total inhibition of cell growth. This indicates that ERK1/2 activity is not only required for mitogen-stimulated proliferation, but is also essential for basal growth. However, activated phospho-ERK1/2 was undetectable in this basal state (Fig 1c), which infers that very low levels of activated ERK1/2, beyond the detection levels of the assay, are facilitating basal levels of proliferation.

### Naïve ADSCs and ADSC-OBs are highly sensitive to inhibition of proliferation by GCs, which is reversed by vanadate

**Fig 3:**
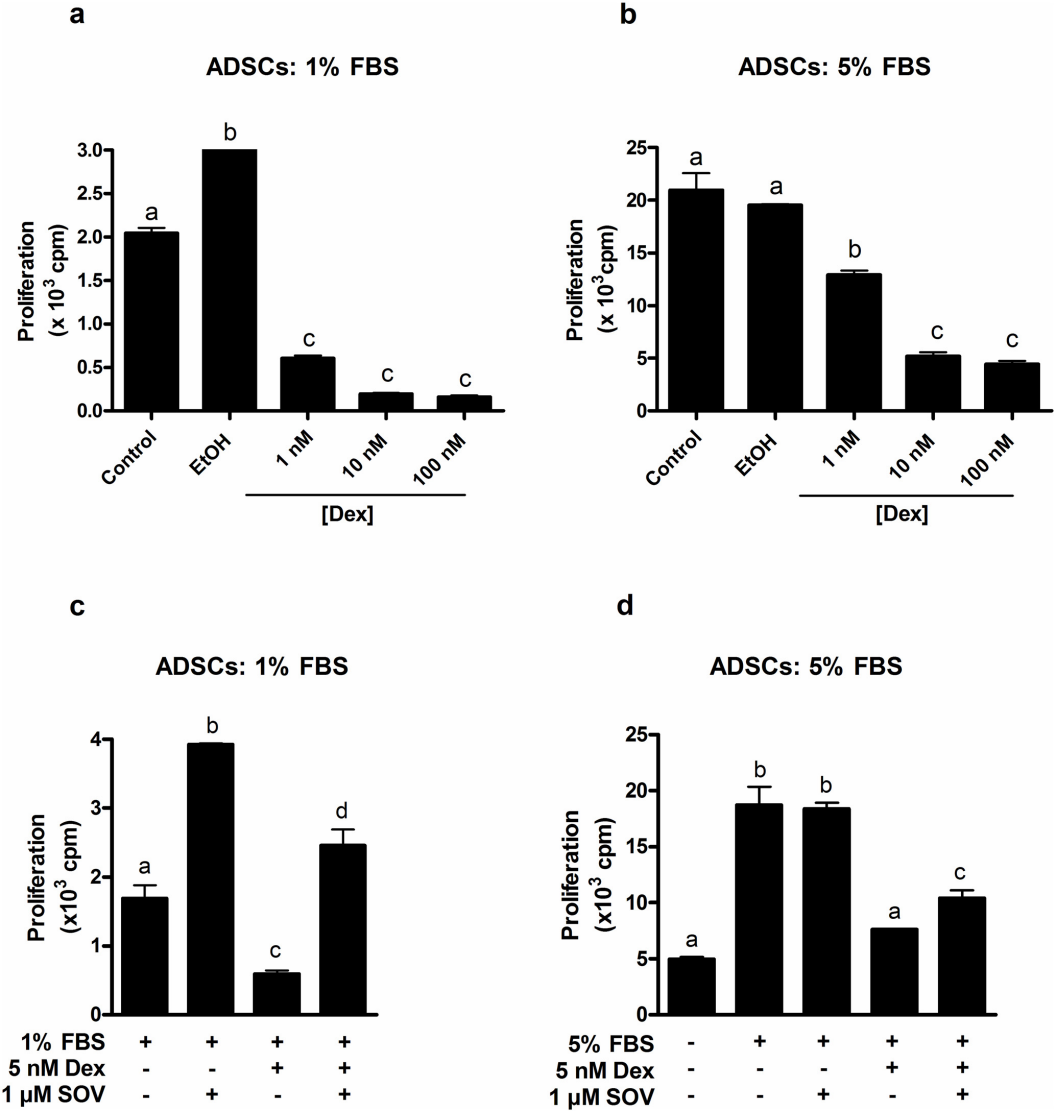
The anti-proliferative effects of GCs on naïve ADSCs are reversed by sodium orthovanadate (SOV). (a-d): Naïve ADSCs were plated into 24-well plates and grown to 80% confluence, after which they were cultured in DMEM + 1% FBS for 24h. (a,b): Cells were subsequently treated for 24h with (a) DMEM + 1% FBS or (b) DMEM + 5% FBS, in the absence (EtOH) or presence of different concentrations of dexamethasone (Dex). (c,d): Cells were subsequently treated for 24h with (c) DMEM + 1% FBS or (d) DMEM + 5% FBS in the absence or presence of 5 nM Dex and 1 μM SOV. For a-d, proliferation was quantified by means of [^3^H]dT incorporation. All panels show the representative results of three independent experiments, performed in triplicate. Different lower-case letters indicate statistically significant differences (P < 0.05).

In order to investigate the effect of GCs on the proliferation of ADSCs and ADSC-OBs, proliferation of these cells was quantified in the absence or presence of increasing concentrations of dexamethasone (Dex). Naïve ADSCs were found to be highly sensitive to proliferative inhibition by Dex, with both background (cells cultured in 1% FBS: Fig 3a) and mitogen-stimulated proliferation (cells in 5% FBS: Fig 3b) substantially reduced at a concentration of 10nM Dex. In order to achieve approximately 50% inhibition of mitogen-stimulated proliferation in naïve ADSCs, a concentration of 5 nM Dex was chosen for subsequent experiments.

The ERK- and other proliferative pathways are negatively regulated by protein tyrosine phosphatases (PTPs) and dual specificity phosphatases (DUSPs) which inactivate key kinases by tyrosine dephosphorylation. Previous work from our group showed that Dex upregulated phosphatase activity in immortalized pre-osteoblasts, and that the anti-proliferative effects of Dex could be reversed by the broad-spectrum PTP inhibitor sodium orthovanadate (SOV) [18], indicating that the upregulated phosphatase activity was directly responsible for the Dex-mediated inhibition of proliferation in these cells. Consequently, we used SOV in the present study to assess whether the GC-induced decrease in proliferation in primary ADSCs and ADSC-OBs was also mediated via PTPs and/or DUSPs. Naïve ADSCs were treated with 5nM Dex, in the absence or presence of 1 μM SOV. Under conditions of basal proliferation (DMEM + 1% FBS), SOV was found to stimulate proliferation and completely abrogated the effect of Dex (Fig 3c). Under conditions of mitogen stimulation (DMEM + 5% FBS), SOV partially reversed the inhibition of proliferation by Dex by approximately 40% (Fig 3d).

**Fig 4:**
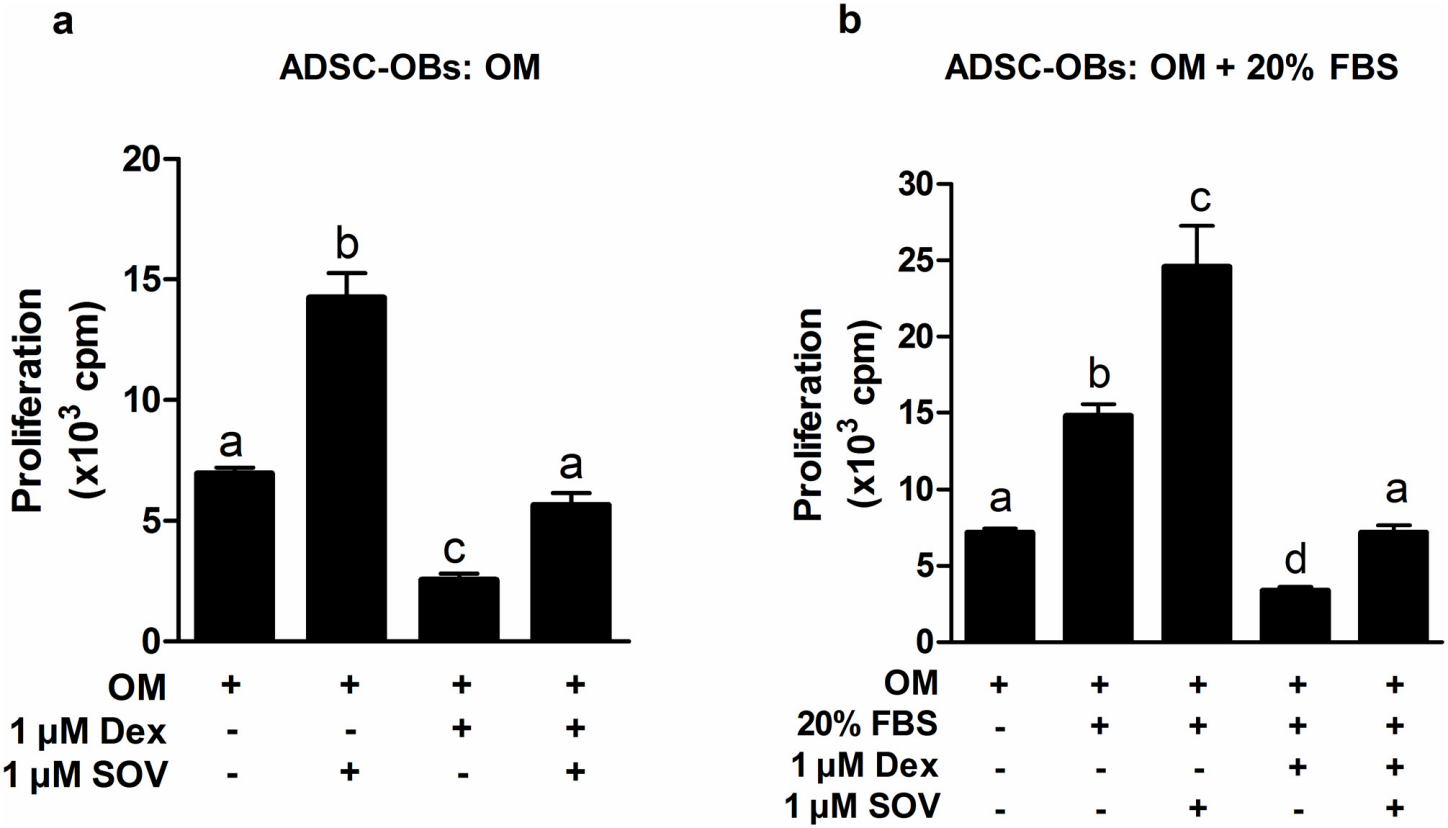
Sodium orthovanadate (SOV) also reversed the anti-proliferative effects of GCs on ADSC-OBs. (a,b): Naïve ADSCs were plated in 24-well plates, grown to 100% confluence and treated with OM for 7 days to generate ADSC-OBs, which were subsequently cultured for 24h in (a) OM + 10% FBS or (b) OM + 20% FBS, in the absence or presence of 1 μM Dex and 1 μM SOV. Proliferation was quantified by means of [^3^H]dT incorporation. All panels show the representative results of three independent experiments, performed in triplicate. Different lower-case letters indicate statistically significant differences (P < 0.05).

In order to study the anti-proliferative effects of GCs on ADSC-OBs, a higher concentration of Dex (1 μM) was used, as a result of standard OM already containing 10 nM Dex. Similar to our findings in naïve ADSCs (Fig 3), 1 μM Dex inhibited basal (OM + 10% FBS: Fig 4a) and mitogen-stimulated (OM + 20% FBS: Fig 4b) proliferation in ADSC-OBs. SOV was found to have mitogenic effects under both sets of conditions (Fig 4a and 4b), and again abrogated the effects of Dex under basal conditions (Fig 4a), while partially reversing the effects of Dex under mitogen-stimulated conditions (Fig 4b).

The reversal of the anti-proliferative effects of Dex by SOV indicates that tyrosine phosphatases, at least in part, contribute to the negative effects of GCs on mitogenesis. However, 1 μM SOV did not totally alleviate the Dex-induced inhibition, suggesting that either PTPs and/or DSPs are not the only conduit for the negative actions of GCs on proliferation, or that 1 μM SOV does not totally inhibit phosphatase activity. Unfortunately, higher concentrations of SOV (10 μM) were found to be cytotoxic, which prevented us from further elucidating the mechanism whereby SOV counteracted the anti-proliferative effects of GCs.

### The effect of GCs on mitogen-stimulated ERK1/2 activation

**Fig 5:**
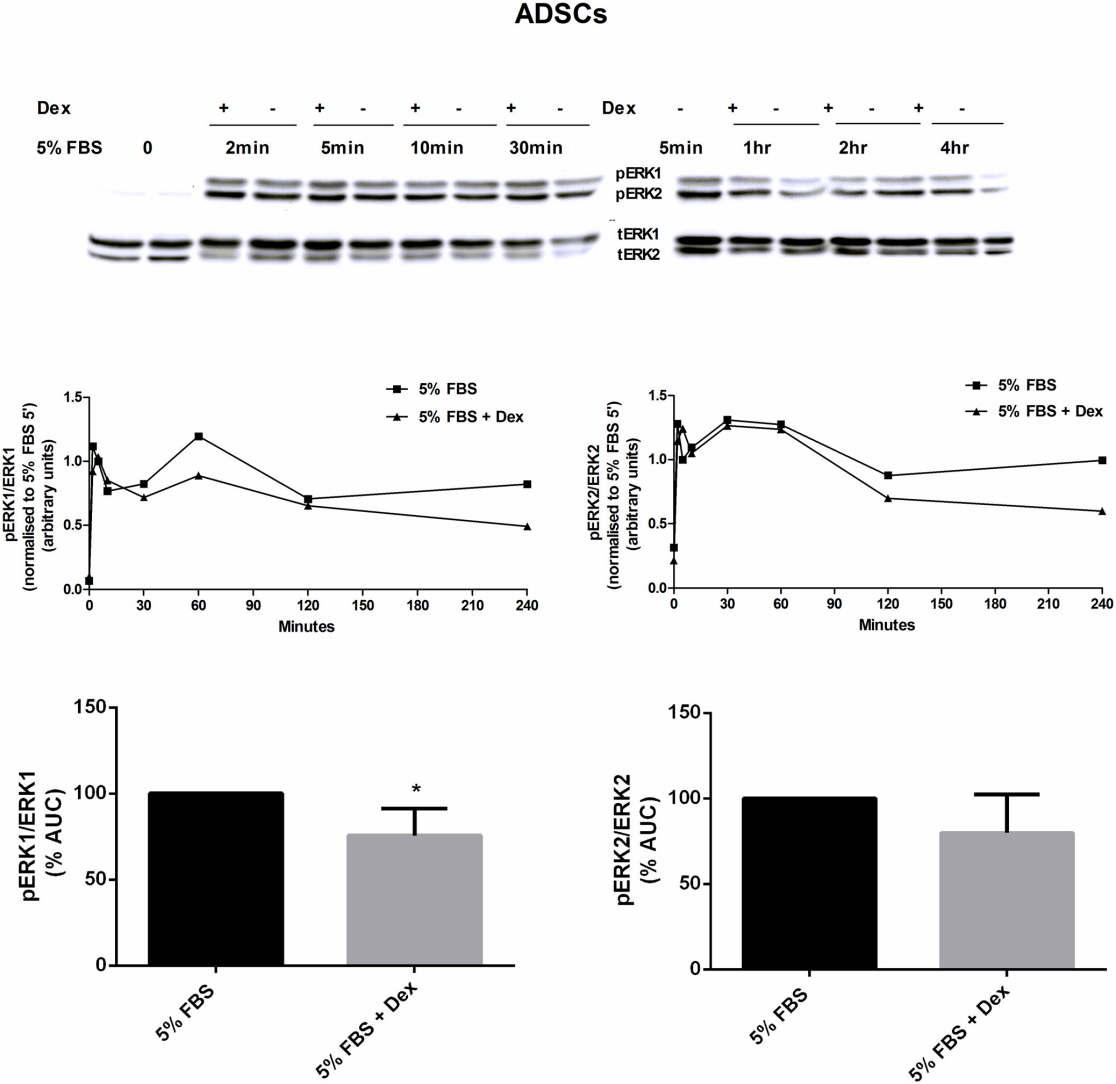
The effect of GC treatment on mitogen-stimulated ERK1/2 activity in ADSCs. Naïve ADSCs were plated in 100mm dishes and grown to 80% confluence, after which they were cultured in DMEM + 1% FBS for 24h. Cells were subsequently pretreated with 10 nM Dex (in the presence of standard growth media: DMEM + 10% FBS) for 1 hour, and then mitogenically stimulated with DMEM + 5% FBS, in the presence of 10 nM Dex (5% FBS + Dex), for the time-points indicated. Control cells were treated with DMEM + 5% FBS (5% FBS). Western blotting for total ERK1/2 and phospho-ERK1/2 was subsequently performed on cell lysates. Immunoresponsive bands were quantified and plotted as the relative intensity of the signal over time, and the area under the curve (AUC) was calculated using Prism software. The representative results of four independent experiments are shown, with * = P < 0.05.

Given our observation that SOV could reverse the anti-proliferative effects of Dex (Figs 3 and 4), we postulated that Dex inhibited proliferation and ERK1/2 activation by upregulating phosphatase expression, as was previously found in MBA-15.4 immortalized pre-osteoblasts [18]. In order to allow for Dex-induced changes in phosphatase gene transcription to occur, Dex treatment was commenced 1 hour before mitogenic stimulation and maintained for the duration of the the mitotic stimulus. Control cells were only subjected to mitogenic stimulation. In naïve ADSCs, 10nM Dex induced only a modest (10–20%) reduction in the mitogen-induced phosphorylation of ERK1 and ERK2 (Fig 5). This reduction was only found to be statistically significant for ERK1.

**Fig 6:**
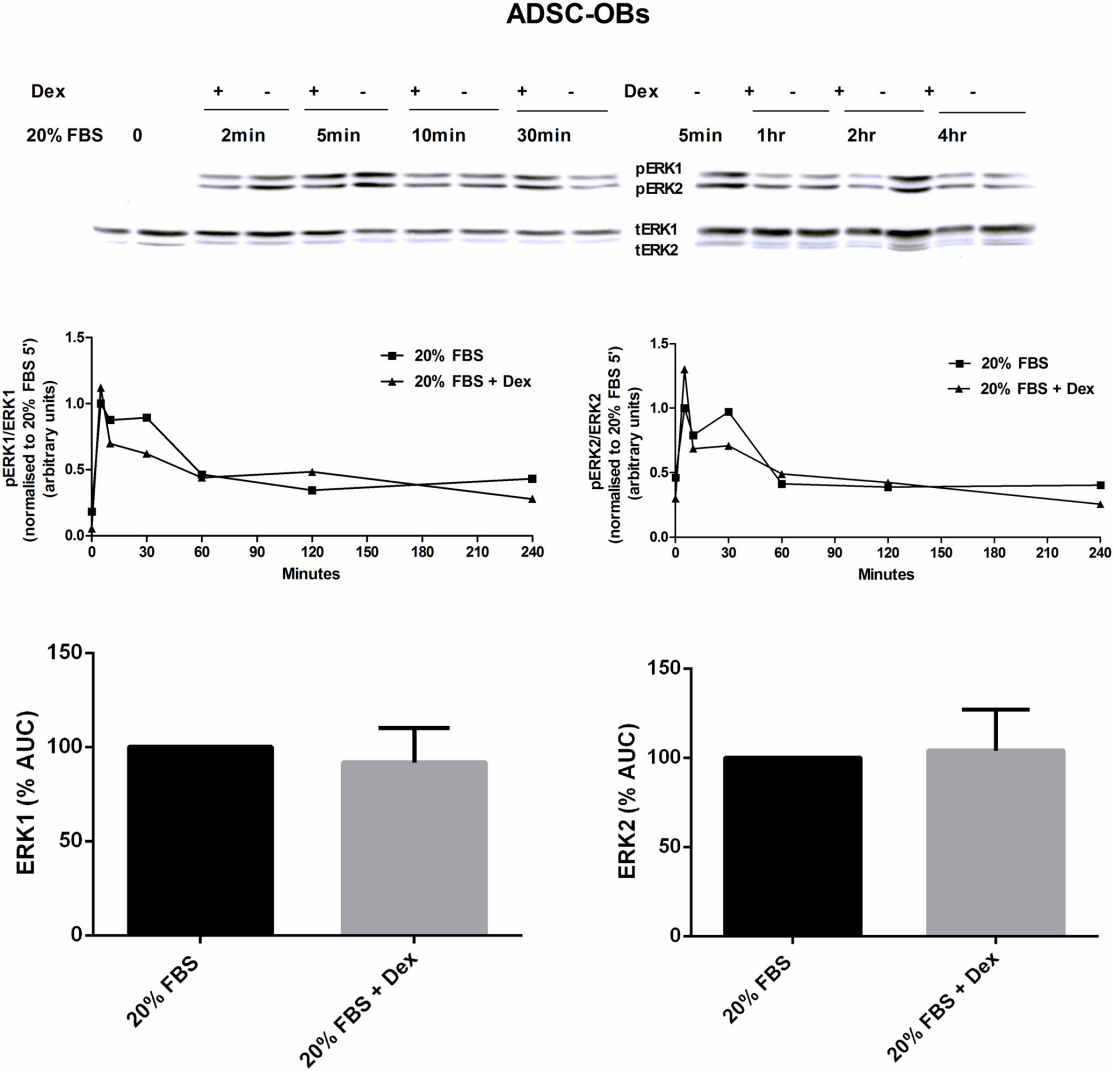
The effect of GC treatment on mitogen-stimulated ERK1/2 activity in ADSC-OBs. Naïve ADSCs were plated in 100mm dishes, grown to 100% confluence and treated with OM for 7 days to generate ADSC-OBs, which were subsequently pretreated with 1 μM Dex (in the presence of OM) for 1 hour, and then mitogenically stimulated with OM + 20% FBS, in the presence of 1 μM Dex (20% FBS + Dex), for the time-points indicated. Control cells were treated with OM + 20% FBS (20% FBS). Western blotting for total ERK1/2 and phospho-ERK1/2 was subsequently performed on cell lysates. Immunoresponsive bands were quantified and plotted as the relative intensity of the signal over time, and the area under the curve (AUC) was calculated using Prism software. The representative results of four independent experiments are shown, with * = P < 0.05.

In ADSC-OBs, the mitogen-induced phosphorylation of ERK1 and ERK2 was largely unaffected by 1 μM Dex treatment (Fig 6).

### The effects of GC treatment on MKP-1 mRNA and protein expression

**Fig 7:**
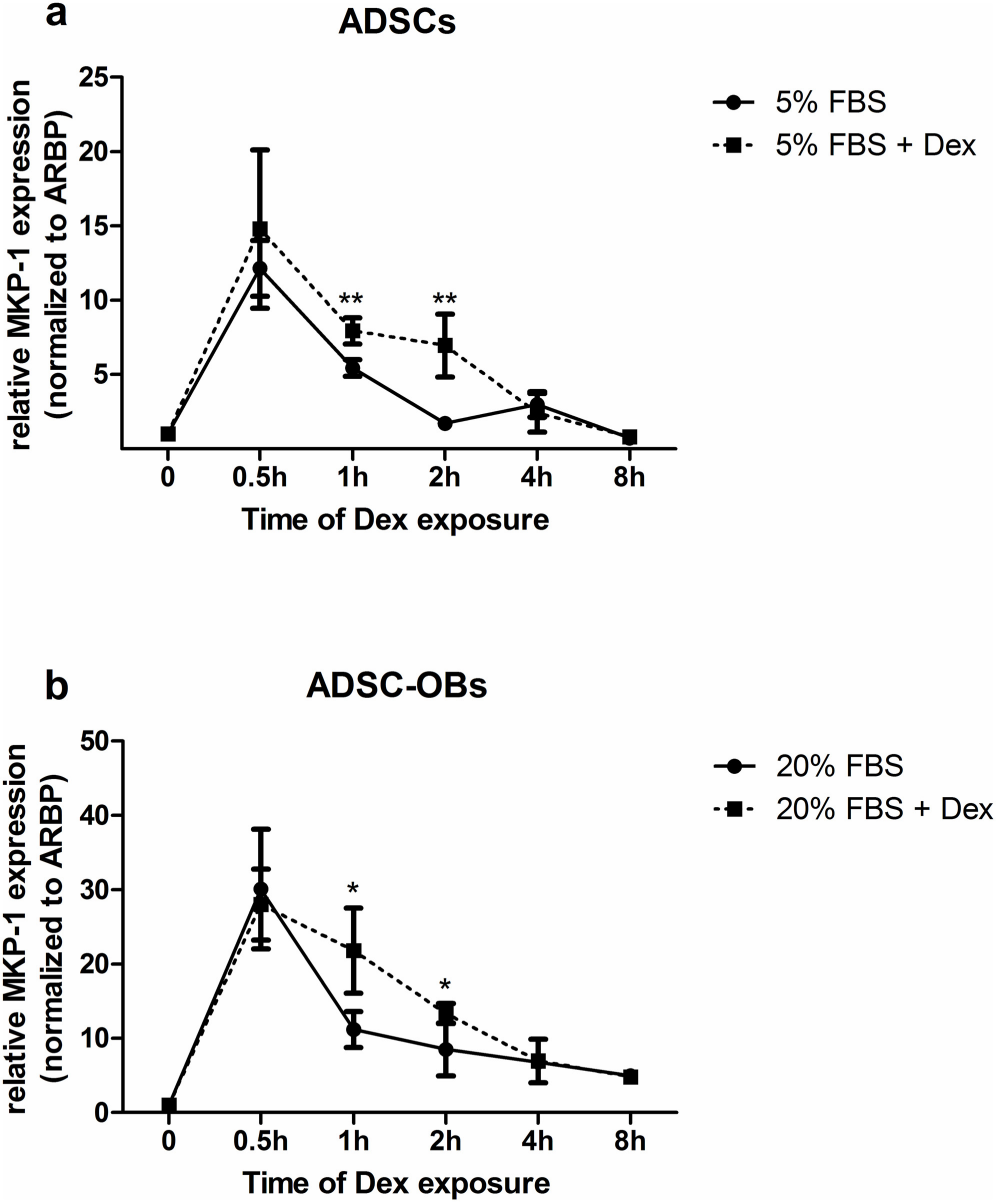
MKP-1 RNA expression is upregulated by mitogenic stimulation and by Dex. (a): Naïve ADSCs were plated in 60mm dishes and grown to 80% confluence before being treated with DMEM + 5% FBS in the absence (solid line) or presence (dotted line) of 1 μM Dex for various time points, after which RNA was isolated. (b): Naïve ADSCs were plated in 60mm dishes, grown to 100% confluence and treated with OM for 7 days to generate ADSC-OBs, which were subsequently treated with OM + 20% FBS in the absence (solid line) or presence (dotted line) of 1 μM Dex for various time points before RNA was isolated. MKP-1 RNA expression levels were quantified by means of qRT-PCR and normalized to expression of the house-keeping gene ARBP. Normalized MKP-1 expression levels in untreated control samples were set as 1. All panels show the representative results of four independent experiments, performed in triplicate. * = P<0.05, ** = P<0.01.

Previous work from our group demonstrated that up-regulation of the MKP-1 gene was critical for the GC-mediated inhibition of proliferation in immortalized pre-osteoblast cell lines [19]. Consequently, using semi-quantitative real-time PCR, MKP-1 gene expression was measured in ADSCs and ADSC-OBs under conditions of mitogenic stimulation, in either the absence or presence of Dex. Mitogenic stimulation resulted in a rapid (< 30 minutes) and significant upregulation of MKP-1 mRNA in both ADSCs (Fig 7a) and ADSC-OBs (Fig 7b). Thereafter, expression levels declined, but partial induction was maintained for at least 8 hours, the last time-point tested. Dex treatment in the presence of mitogenic stimulation resulted in a more pronounced upregulation of MKP-1 mRNA levels in ADSCs and ADSC-OBs at 1h and 2h (Fig 7a and 7b), but this effect was lost at later time points (4h and 8h). Due to the cytotoxic effects of Dex in these cells against the background of 1% FBS, the effects of Dex on MKP-1 expression in the absence of mitogenic stimulation could not be determined.

**Fig 8:**
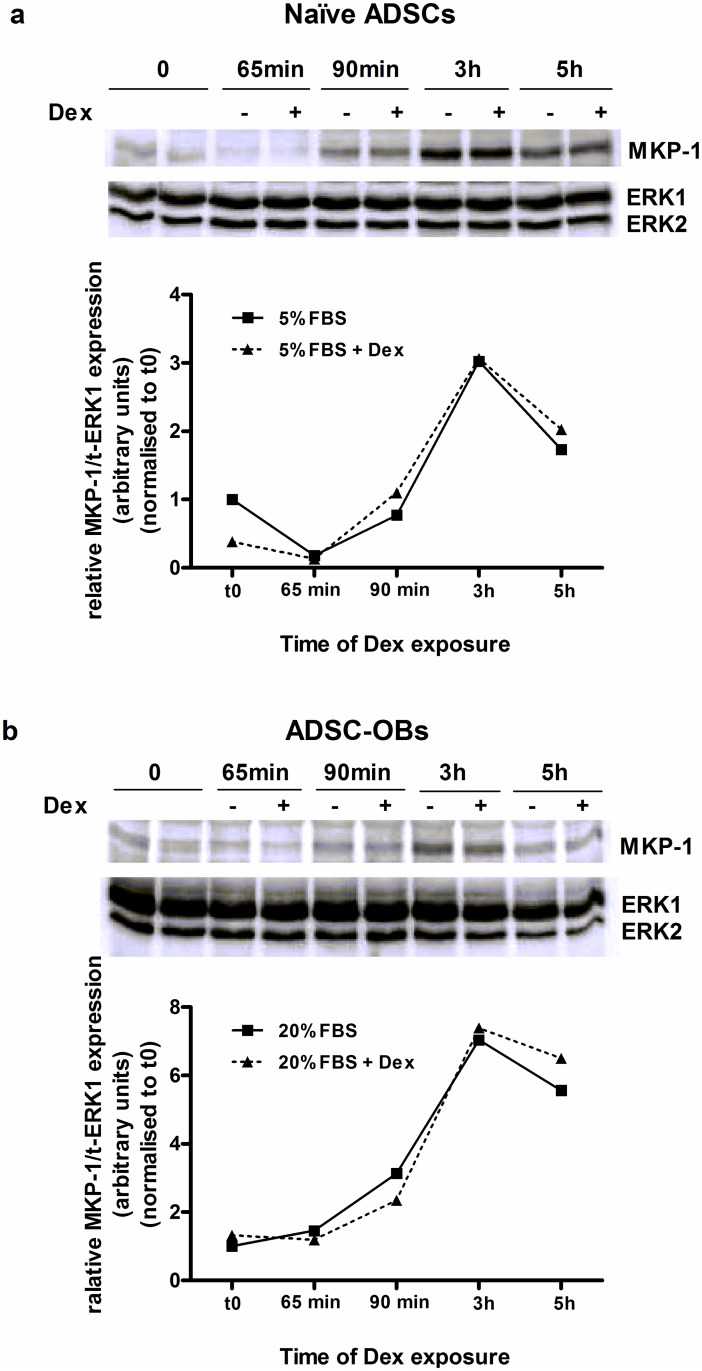
MKP-1 protein expression is upregulated by mitogenic stimulation, but unaffected by Dex. (a): Naïve ADSCs were plated in 100mm dishes and grown until 80% confluence, after which they were pre-treated for 1 hour with 1 μM Dex (in standard growth media: DMEM + 10% FBS) and subsequently mitogenically stimulated with DMEM + 5% FBS, in the presence of 1 μM Dex, for 5 minutes, 30 minutes, 2 hours and 4 hours (5% FBS + Dex). Control cells were treated with DMEM + 5% FBS for 5 minutes, 30 minutes, 2 hours and 4 hours (5% FBS). (b): Naïve ADSCs were plated in 100mm dishes, grown until 100% confluence and with OM for 7 days to generate ADSC-OBs, which were pre-treated for 1 hour with 1 μM Dex (in OM) and subsequently treated with OM + 20% FBS, in the presence of 1 μM Dex (20% FBS + Dex), for the time-points mentioned in (a). Control cells were treated with OM + 20% FBS for the time-points mentioned in (a). Cell lysates were prepared and Western blotting for MKP-1 and total ERK1/2 was performed. MKP-1 protein expression levels (normalized to total ERK1) in untreated control samples were set as 1. All panels show the representative results of four independent experiments.

Western blot analysis revealed that mitogenic stimulation also upregulated MKP-1 protein levels in naïve ADSCs (Fig 8a) and ADSC-OBs (Fig 8b), peaking at 3 hours post-stimulation in both cell-types. However, Dex treatment had no effect on the mitogenic induction of MKP-1 protein expression (Fig 8a and 8b).

## Discussion

The results from the present study indicate that mitogenic stimulation in primary naïve ADSCs and ADSC-derived pre-osteoblasts (ADSC-OBs) is mediated via the classical ERK1/2 pathway. Both naïve ADSCs and ADSC-OBs were found to be sensitive to the inhibitory effects of GCs (Dex) on basal and mitogen-induced cellular proliferation, which could be reversed by the broad-spectrum PTP/DUSP inhibitor SOV. However, Dex treatment had only a small inhibitory effect on mitogen-stimulated ERK1/2 activity, whereas protein expression levels of MKP-1, a DUSP that was previously shown to mediate the inhibitory effects of GCs on ERK1/2 activity and proliferation in immortalized pre-osteoblasts [18,19], were not affected by GCs in either primary naïve ADSCs or ADSC-OBs.

Our experiments examining the anti-proliferative effect of GCs demonstrated that 5 nM Dex was sufficient to induce an approximately 50% decrease in mitogen-stimulated proliferation in naïve ADSCs, while 1 μM Dex was required to reduce mitogen-stimulated proliferation in ADSC-OBs by approximately 75%, indicating that naïve ADSCs are considerably more sensitive to Dex than ADSC-OBs. Earlier results from our group demonstrated a similar desensitization to Dex in ROB-C26 mature immortalized osteoblasts, compared to MBA 15.4 immortalized early pre-osteoblasts [6]. These findings correspond to results from studies in cultured primary fetal calvarial osteoblasts showing that sensitivity to Dex decreases as cells progress towards a mature osteoblastic phenotype [30]. In addition, 100 nM Dex was found to be required for 25–45% inhibition of mitogen-stimulated proliferation in MBA 15.4 immortalized early pre-osteoblasts [18], indicating that primary ADSCs are between 10- and 20-fold more sensitive to GC-induced growth retardation compared to immortalized cells, which may be the result of the corrupted cell cycle in immortalized cells that robustly maintains proliferative capacity for perpetual growth.

In the present study, it was found that 24h treatment with 5% FBS resulted in maximal mitogenic stimulation of naïve ADSCs, whereas 20% FBS was required for mitogenic stimulation of ADSC-OBs. This difference is most likely a function of the proliferative capacity of these cells, with naïve ADSCs being highly proliferative, while the proliferative index of differentiating ADSC-OBs was diminished, as has been described before [31] and therefore required higher concentrations of growth factors in order to be stimulated. The inhibition of ERK1/2 activation by U0126 resulted in the near total abrogation of mitogen-stimulated and background proliferation, indicating that ERK1/2 activation is essential for mitogenesis. Given that basal proliferation (in the absence of mitogenic stimulation) is further reduced by U0126, it is likely that basal ERK1/2 activity may, at the very least, be permissive for proliferation to occur and may actively promote mitogenesis. However, this does not exclude the possibility that the modulation of other signalling pathways may also be required to be activated for mitogen-induced and basal proliferation.

Although our results with U0126 demonstrated the essential requirement of ERK1/2 activation for proliferation in naïve ADSCs and ADSC-OBs, we found that GCs strongly inhibited FBS-induced proliferation in these cells, while having very little effect on FBS-induced ERK1/2 activity. This is similar to our earlier observations in MBA15.4 immortalized pre-osteoblasts, where a relative small Dex-induced reduction in MAPK activity resulted in a disproportionate decrease in mitogen-stimulated proliferation [6]. In order to reconcile these apparently conflicting findings, we postulate that mitogenic stimulation in ADSCs and ADSC-OBs requires ERK1/2 activation in order to allow proliferation to proceed, but also involves the modulation of another, unidentified, pathway that is sensitive to inhibition by GCs without affecting the phosphorylation status of ERK1/2. Although outside of the scope of the present study, this possibility warrants further investigation.

Earlier work from our group demonstrated that the DUSP MKP-1 was upregulated in response to GCs in immortalized MBA15.4 and MG-63 pre-osteoblasts [18], and that MKP-1 expression and activity were critical for the GC-mediated inhibition of proliferation in these pre-osteoblast cell lines [19]. However, subsequent experiments in MKP-1 null mice found that these animals were just as susceptible to the osteoporotic effects of GCs as their wild-type litter-mates [20], indicating that MKP-1 was not absolutely required for mediating the pathological effects of GCs on bone *in vivo*. In order to reconcile these conflicting findings from MKP-1 null mice and immortalized pre-osteoblasts, we sought to examine the expression of MKP-1 in primary ADSCs, which are generally regarded to be more representative of the behaviour of cells *in vivo* than immortalized cell lines. In the present study, we found a strong upregulation of MKP-1 mRNA and protein expression, both in naïve ADSCs and in differentiating ADSC-OBs, in response to mitogenic stimulation. However, this upregulation was unaffected by GCs, suggesting that MKP-1 is not involved in mediating the anti-proliferative actions of GCs in these cells. While GC treatment against the background of mitogenic stimulation resulted in a stronger upregulation of MKP-1 mRNA after 1–2 hours than what was observed for mitogenic stimulation alone, this was not correlated with an increase in protein levels, and the biological significance of this increased mRNA expression is therefore unclear. Although these findings do not completely preclude a role for MKP-1 for mediating some of the effects of GCs on osteoblast precursor cell proliferation, it seems unlikely that MKP-1 is the sole or predominant mediator of the anti-proliferative effects of GCs in these cells. This is in contrast to our findings in MBA-15.4 cells [19], and may possibly be a result of the cell cycle dysregulation in immortalized cells. The cell cycle is exquisitely regulated *in vivo* and *ex vivo* in primary cells, to insure an appropriate response to mitogenic signals from the extracellular milieu. However, in immortalized cell lines this regulation has been corrupted in order to perpetually propagate these cells and therefore it is possible that mitogenic responses in these cells may differ mechanistically from those *in vivo* or in primary cells.

The results from the present study are in accordance with our findings in MKP-1 null mice [20], and indicates that either MKP-1 may not mediate the actions of GCs in bone, or that there is degeneracy in this process, with one or more other GC-responsive proteins being able to reduce osteoblast numbers and consequently bone density. Evidence for degeneracy in the ERK/MKP-1 pathway is provided by the observation that MKP-1 null mice are phenotypically and histologically normal, and that ERK activity in MKP-1^-/-^ mouse embryo fibroblasts is unaltered, suggesting that other phosphatases can compensate for the absence of MKP-1 [32]. These observations, together with our own *in vivo* [20] and *ex vivo* findings described here, led us to conclude that no additional insight would be gained by repeating our *ex vivo* study in cells derived from MKP-1 null mice, especially since a primary point of interest in the present study was the regulation of endogenous MKP-1 expression by Dex. Aside from degeneracy, it is also possible that MKP-1 is simply not involved in mediating the anti-proliferative effects of GCs in bone cells. There are currently 10 active MKPs identified, with some degree of substrate specificity for particular MAP kinases [33,34]. Many MKPs are inducible or early-response genes [35] and are often induced by the same stimuli that activate MAP kinases, especially mitogens [36], as was observed in the present study. MKP-1 was one of the first MKPs to be identified, and it is noteworthy that MKP-1 preferentially dephosphorylates p38, with a lesser affinity for ERK1/2 [34]. Correspondingly, the majority of the *in vivo* functions of MKP-1 described to date appear to be mediated through the modulation of p38 activity [37–40]. In contrast, the MKPs MKP-3/DUSP6, MKP-X/DUSP7 and DUSP5 all specifically target ERK1/2 [33,34], although the possible involvement of these MKPs in mediating the osteoporotic effects of GCs has not been investigated in detail. Furthermore, as Dex treatment of ADSCs and ADSC-OBs in the present study did not result in a pronounced dephosphorylation of ERK1/2, it may be speculated that the anti-proliferative effects of GCs in these cells occur independent of Dex-responsive MKPs. Our results suggest that kinases other than ERK1/2 are also involved in mitogen-stimulated proliferation, and consequently other phosphatases could act as conduits for the anti-proliferative effects of GCs. A more detailed study would therefore be required to elucidate the mechanism whereby GCs inhibit proliferation in primary ADSCs and ADSC-derived pre-osteoblasts.
